# Graphene microfiber as a scaffold for regulation of neural stem cells differentiation

**DOI:** 10.1038/s41598-017-06051-z

**Published:** 2017-07-18

**Authors:** Weibo Guo, Jichuan Qiu, Jingquan Liu, Hong Liu

**Affiliations:** 1 Shandong Province Key Laboratory of Detection Technology for Tumor Makers, College of Chemistry and Chemical Engineering, Linyi University, Linyi, 276005 P. R. China; 2 State Key Laboratory of Crystal Materials, Shandong University, Jinan, 250100 P. R. China; 3 College of Materials Science and Engineering, Laboratory of Fiber Materials and Modern Textile, The Growing Base for State Key Laboratory, Qingdao University, Qingdao, 266071 P. R. China

## Abstract

We report the cytocompatibility and regulating effects of the nanostructured reduced graphene oxide (rGO) microfibers, which are synthesized through a capillary hydrothermal method, on neural differentiation of neural stem cells (NSCs). Our findings indicate that the flexible, mechanically strong, surface nanoporous, biodegradable, and cytocompatible nanostructured rGO microfibers not only offer a more powerful substrate for NSCs adhesion and proliferation compared with 2D graphene film and tissue cluture plate but also regulate the NSCs differentiation into neurons and form a dense neural network surrounding the microfiber. These results illustrate the great potential of nanostructured rGO microfibers as an artificial neural tissue engineering (NTE) scaffold for nerve regeneration.

## Introduction

Nerve injury is a common global health problem which could significantly affect patients’ quality of life and cause an enormous socioeconomic burden^1^. Due to the limited regenerative capabilities of the nervous system, stem-cell-based NTE has shown great potential for nerve regeneration by taking advantage of the self-renewal and differentiation capabilities of stem cells^2^, such as mesenchymal stem cells (MSCs)^3^ and NSCs^4^. A microscale fiber-shaped structure, which can act as a physical graft for axon growth between the nerve sites, is one of the most typical biological components in nerve tissue^5^. A wide variety of synthetic polymers and natural polymers have been evaluated for use as NTE fiber-shaped scaffolds^6^. The major drawbacks of the natural material based scaffolds are that they do not possess sufficient mechanical stability and have a tendency to collapse after implantation^7^. Some synthetic polymers have advantages over natural materials, for example, the degradation speed can be tuned by altering their molecular weight and composition. However, the potential drawbacks of synthetic polymers are not ignorable, including the loss of mechanical stability after implantation and the release of acidic degradation products^8^. Therefore, designing microscale fiber-shaped scaffolds from ideal biomaterials is still an ongoing challenge due to the need to overcome the above-mentioned drawbacks.

Graphene is a two-dimensional (2D) monolayer sheet of sp^2^-hybridized carbon atoms packed in a zero-band honeycomb lattice^9^. Owing to the unique chemical and physical properties and its biocompatibility, graphene has attracted much attention for numerous potential applications in biotechnology, such as biosensing^10^, disease diagnostics^11^, antibacterial functionality^12^, cancer targeting^13^, photothermal therapy^14^ and electrical stimulation electrodes^15^. Graphene oxide (GO) is a type of graphene nanosheet with many oxygen-containing groups (such as -OH and -COOH), which can be produced through a chemical exfoliation method from bulk graphite. GO can be reduced to reduced graphene oxide (rGO), which could restore the physical and chemical properties of graphene. GO and rGO scaffolds possess great biocompatibility and bioactivity^16^, but the poor plasticity is the main limitation for its NTE applications^17^.

In this paper, a nanostructured rGO microfiber was synthesized by a dimensionally confined hydrothermal reduction strategy, which presents the possibility for the application of rGO in NTE *in vitro* ^18^. By assessing the cytocompatibility and differentiation of NSCs on the nanostructured rGO microfiber, the adhesion and proliferation of NSCs were studied, and the regulating effects of the differentiation of NSCs to neurons were proven. This work suggests that the nanostructured rGO microfiber has great cytocompatibility, unique structure and nanoporous topography, which would benefit the differentiation of NSCs, especially for neural differentiation. Our work opens a way to realize the highly efficient neural differentiation of NSCs that will provide potential opportunities in nerve regeneration.

## Results and Discussion

### Characterization of nanostructured rGO microfiber

**Figure 1:**
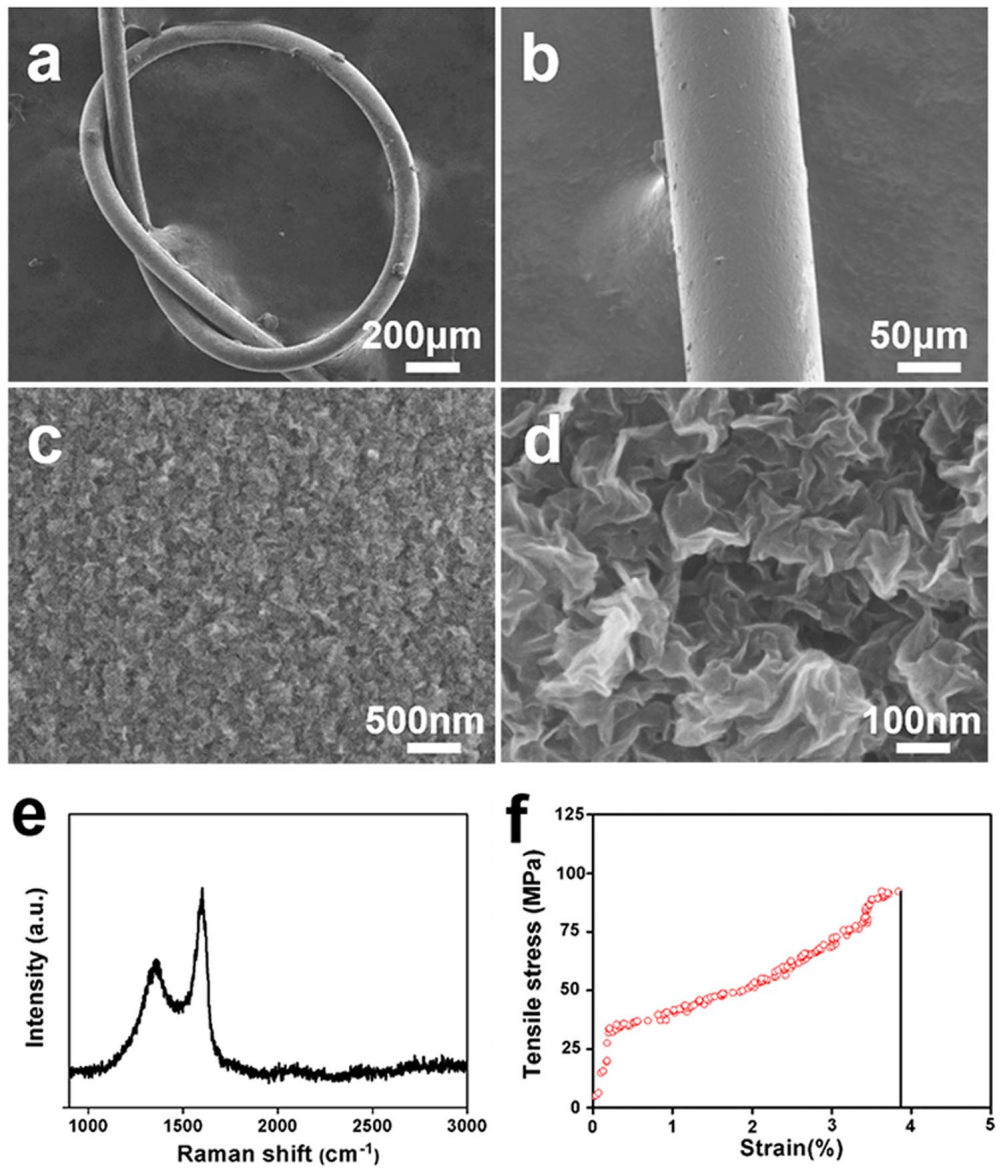
Characterization of nanostructured rGO microfiber. (**a**) SEM image of a knotted nanostructured rGO microfiber, (**b**) SEM image of a straight nanostructured rGO microfiber, and (**c**) (**d**) high-resolution SEM image of the surface morphology of the nanostructured rGO microfiber. (**e**) Raman spectrum of a nanostructured rGO microfiber (black curve), (**f**) stress-strain curve of an as-prepared nanostructured rGO microfiber (red circle).

GO was prepared by the modified Hummers’ method as described in our previous work^3^, the GO nanosheets used for the preparation of the nanostructured rGO microfibers are approximately 1.5 nm thick and range from 1~4 μm in size (Fig. S1). The morphology and microstructure of the as-prepared nanostructured rGO microfiber were observed by scanning electron microscope (SEM), from Fig. 1a, the diameter of the nanostructured rGO microfiber is approximately 100 μm, and the microfiber shows good flexibility. For example, it is easily knotted without any fracturing or cracking. The wire morphology and flexibility endow the nanostructured rGO microfiber with potential application as a NTE scaffold.

Figure 1b shows an rGO fiber with a uniform diameter of 100 μm. The high-resolution SEM images (Fig. 1c,d) show the specific morphology of the surface of the rGO fiber. Figure 1c indicates that the rGO fiber has a rough surface morphology. From Fig. 1d, we can find that the microfiber is composed of graphene nanosheets. The bended nanosheets are connected to each other forming a 3D porous nanostructure, and the size of the pores range from 100~500 nm, the whole microfiber is composed of the 3D rGO nanostructures. Therefore, the fiber is a nanostructured rGO microfiber. The flexibility of the rGO nanosheets and the kinked morphology of the interconnected graphene sheets endow the nanostructured rGO microfiber with excellent flexibility and tensile stress. The crystalline structure of the graphene nanosheets was characterized by Raman spectroscopy, two characteristic D-band and G-band peaks can be observed at ~1350 cm^−1^ and ~1590 cm^−1^, respectively (Fig. 1e). The calculated ratio of the intensity of the D band to G band is approximately 0.66, and about 1.0 in wet rGO microfiber (Fig. S3), indicating a good alignment of rGO nanosheets in nanostructured rGO microfiber^19^. Zeta potential and O/C ratio are also important parameters to evaluate the performance of graphene-based scaffolds in cell culture. As shown in Figs S4, S5, the zeta potential of rGO is about −10.58 ± 1.83 mV and the O/C ratio of the rGO surface is about 0.89. The results indicate that the surface of nanostructured rGO microfiber is negative charged and containing abundant oxygen-containing groups, which are beneficial for cell adhesion^20^. The dry rGO fibers have an average density of 0.24 g/cm^3^ ^21^, which is approximately 7 times lower than that of the carbon fibers (1.7~2.0 g/cm^3^)^22^. A tensile test of the nanostructured rGO microfiber was performed, and the result (Fig. 1f) shows that the tensile strength of the nanostructured rGO microfiber was over 90 MPa at room temperature, indicating that the microfiber possesses light weight and suitable mechanical properties. The protein adsorption ability of the nanostructured rGO microfiber was also assessed (Fig. S6). Compared with 2D graphene film (Fig. S8), BCA quantitative measurement indicates that the amount of adsorbed bovine serum albumin (BSA) on rGO microfibers was about 11.88 μg and 5.53 μg on graphene film. The BSA adsorption amount on nanostructured rGO microfibers is about 2.15-fold higher than that on 2D graphene film, showing the nanostructured rGO microfiber could be more efficient for cellular adhesion. The biodegrability of nanostructured rGO microfibers was also described in our previous work^15^. These results show that the nanostructured rGO microfiber is a potential candidate for NTE scaffolds.

### Cytocompatibility, proliferation rate, adhesion and spreading of NSCs on the substrates

**Figure 2:**
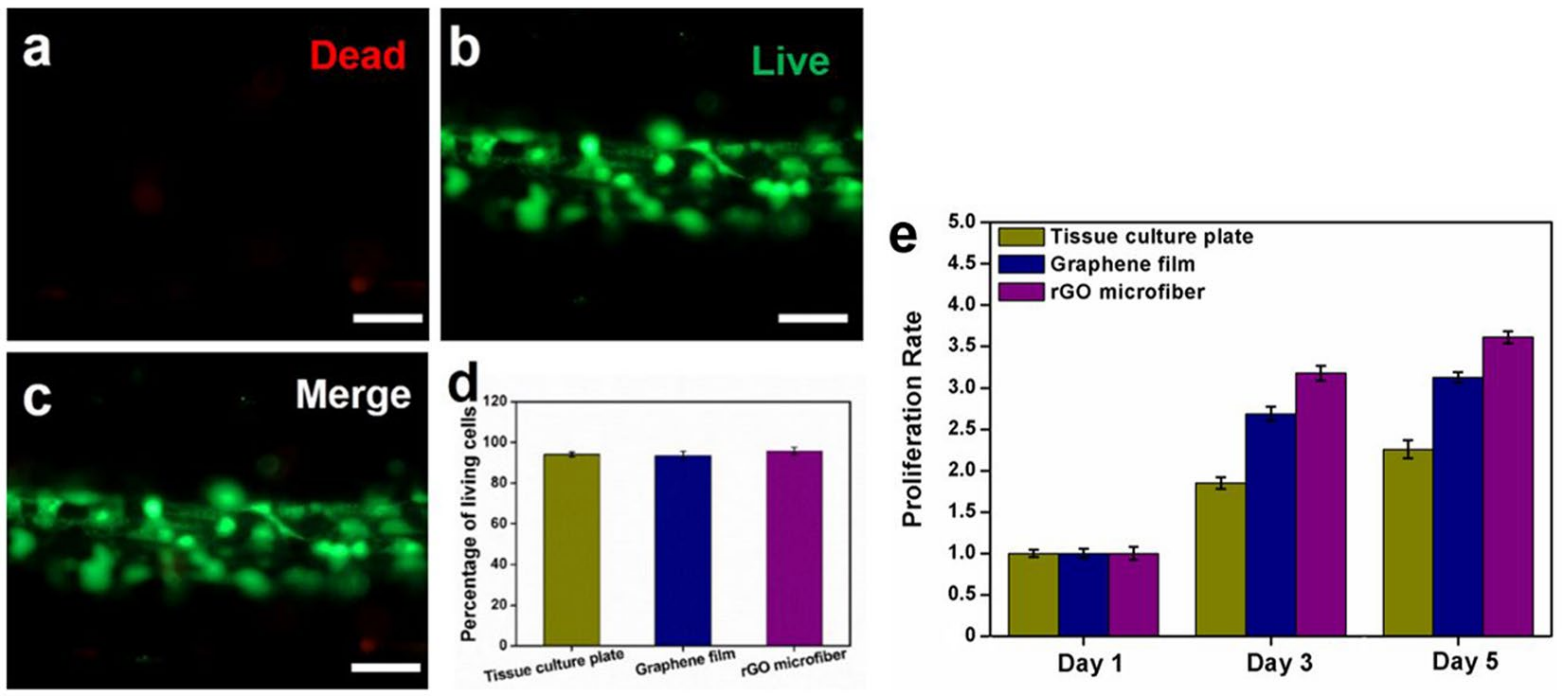
NSCs viability on the scaffolds. Cell viability assay of NSCs on the nanostructured rGO microfiber after 3 days of culture by the Live/Dead assay; dead cells were stained red (**a**), live cells were stained green (**b**), and (**c**) is the merged image; (Scale bar = 50 μm) the percentage of living cells (**d**) and cells proliferation rate (**e**) on tissue culture plate, 2D graphene film and nanostructured rGO microfiber.

Due to Nestin is a key identifier for an immature stem or progenitor phenotype, the purity of the isolated NSC spheres were assessed by Nestin staining, as shown in Fig. S7. The result illustrates that the isolated NSCs have high purity. The cytotoxicity of the nanostructured rGO microfiber was evaluated by a Live/Dead cellular staining assay after the NSCs were cultured on microfibers for 3 days under proliferation conditions with tissue culture plates and 2D graphene films as controls (as shown in Figs S9 and S10). Dead cells were stained red (Fig. 2a), and live cells were stained green (Fig. 2b). The merged Fig. 2c shows that ~95.8% of the NSCs are alive after culturing on the nanostructured rGO microfiber, while ~94.1% on tissue culture plate and ~93.5% on 2D graphene film.

The comparative proliferation rates of NSCs on the tissue culture plate, 2D graphene film and nanostructured rGO microfiber were determined by a CCK-8 cells proliferation assay on the first, third, and fifth day after cells seeding (Fig. 2e). On the third and fifth day, NSCs proliferation rates on the graphene substrates were significantly higher than those on the tissue culture plate, in the meanwhile, the cells proliferation rate of NSCs on the nanostructured rGO microfiber was higher than that on the 2D graphene film, these results indicated that the graphene substrates have positive effects on cell attachment and spreading compared with the tissue culture plate, which mainly caused by the higher protein adsorption abilities. This further suggested that the nanostructured rGO microfibers which process best protein adsorption ablity could be used as a cytocompatible platform for NSCs.

**Figure 3:**
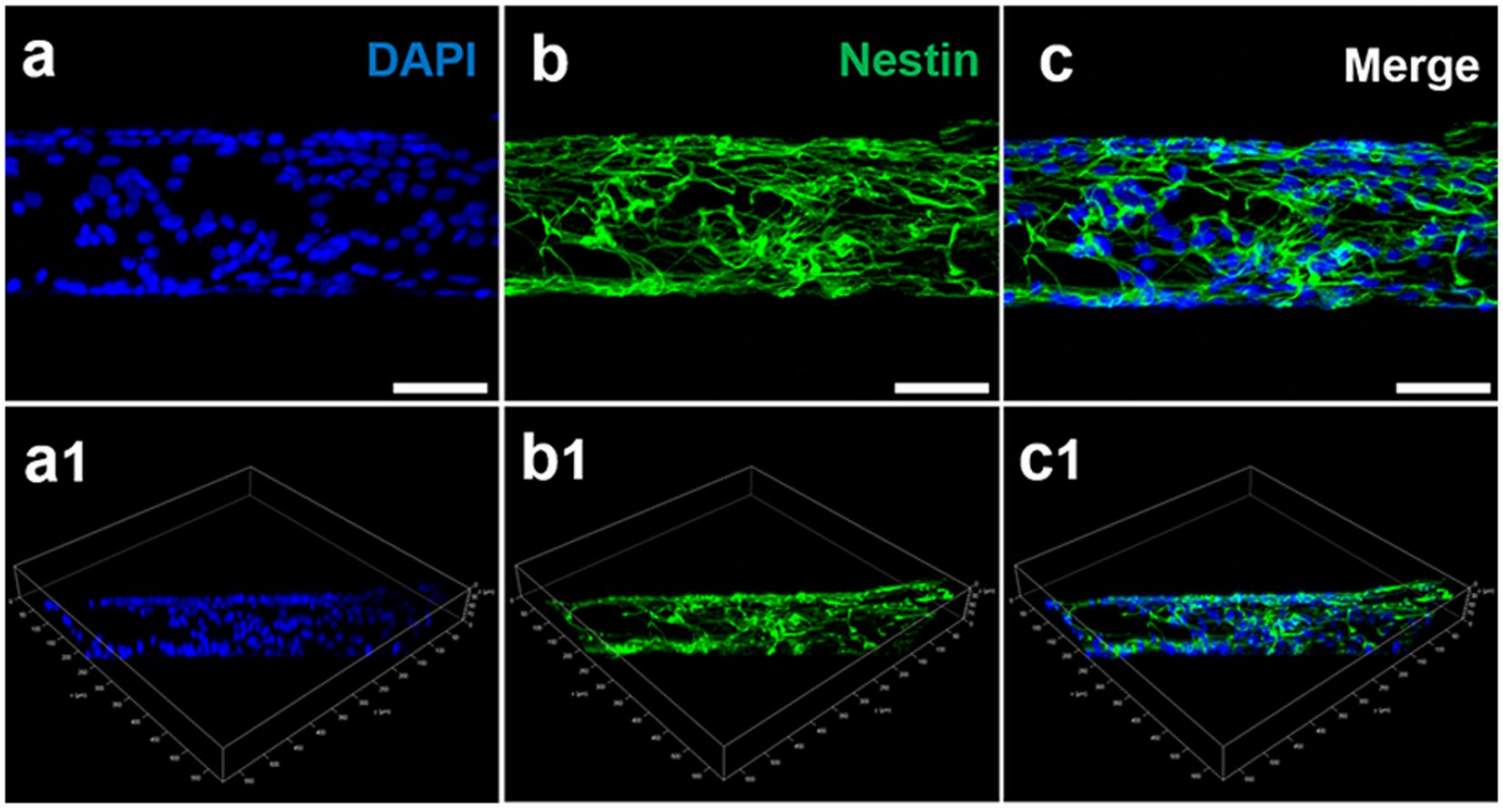
NSC adhesion on nanostructured rGO microfiber. 2D CLSM fluorescence micrographs of NSCs proliferated on the nanostructured rGO microfiber for 5 days; immunostaining makers were DAPI (blue) (**a**) for nuclei and Nestin (green) (**b**) for NSCs, and their images were merged (**c**). (Scale bar = 50 μm) The 3D structure CLSM fluorescence micrographs of the nanostructured rGO microfiber with DAPI (**a1**) and Nestin (**b1**) stained NSCs are also presented; (**c1**) is the merged micrograph.

By the immunostaining of Nestin (Fig. 3b), the cells adhesion on the surface of the nanostructured rGO microfiber was investigated. From Fig. 3c, we find that the fluorescent cells distribute as a wire shape, which matches the diameter and morphology of the nanostructured rGO microfiber. The wire-like cells connect to each other to form a network, which is the typical immature phenotype of NSCs. As controls, NSCs were also cultured on tissue culture plate (Fig. S11a) and graphene film (Fig. S11b). From the fluorescence images, cells have a higher density and attachment on the nanostructured rGO microfiber, which may be due to the better protein adsorption. What should be particularly noted is that the NSCs can grow preferentially around the graphene fiber, establishing a cells network on the surface of the nanostructured rGO microfiber. Movie S1 and its 3D images shown in Fig. 3(a1, b1 and c1) suggest that NSCs uniformly attach on the surface of the nanostructured rGO microfiber and formed a tubular structure on the surface of the nanostructured rGO microfiber. The above results prove that the nanostructured rGO microfiber not only possesses great cytocompatibility for NSCs but also can enhance the adhesion of NSCs, which was mainly because of the better protein adsorption ability of the microfiber.

**Figure 4:**
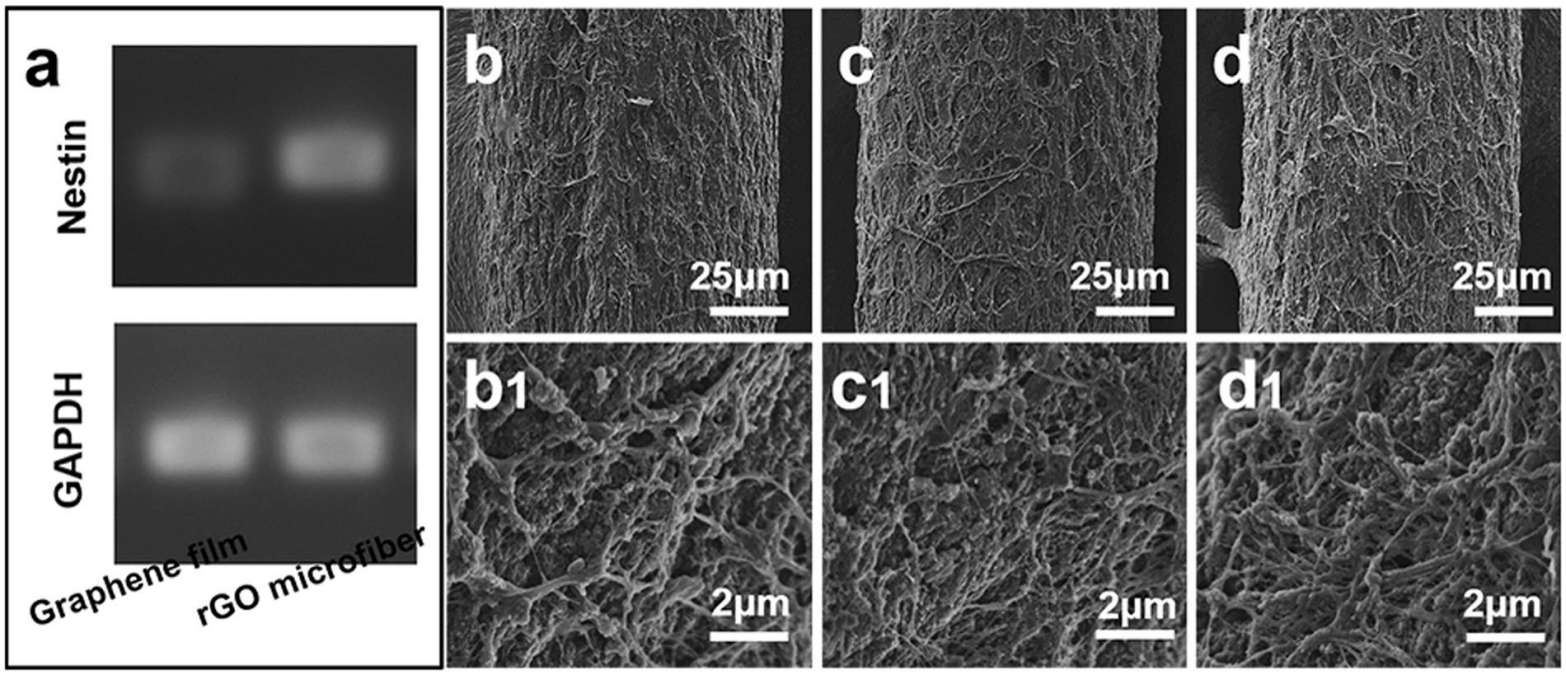
NSCs spreading on nanostructured rGO microfiber. (**a**) Nucleic acid electrophoresis analysis of Nestin expression on 2D graphene film and nanostructured rGO microfiber after 3 days culture. NSCs spreading on the nanostructured rGO microfiber, SEM images of the morphologies of NSCs cultured on nanostructured rGO microfibers for (**b**) 5, (**c**) 10 and (**d**) 15 days, and (**b1**), (**c1**), (**d1**) are their high-resolution SEM images, respectively.

After assessing the cytocompatibility of the nanostructured rGO microfibers, the adhesion property of NSCs on the microfibers was detected. The quantitative cDNA nucleic acid electrophoresis analysis of Nestin expression (GAPDH as control) on 2D graphene film and nanostructured rGO microfiber after 3 days culture illustrates that the expression of Nestin is significantly higher on nanostructured rGO microfibers than that on 2D graphene film, suggesting that a higher percentage of cells were Nestin positive, it seems more cells on nanostructured rGO microfiber maintaining the immature phenotype compared to the 2D graphene film (Fig. 4a). The morphologies of NSCs spreading on the nanostructured rGO microfibers at different times were observed by SEM. After 5 days, there is a layer of cells on the surface of the nanostructured rGO microfiber (Fig. 4b), the cells covered almost 50% area of the fiber surface. At day 10 (Fig. 4c), a significant difference in the morphologies of NSCs coverage on the nanostructured rGO microfiber was observed, approximately 80% of the area is covered by the elongated neurites, and more networked cells can be found. At day 15, leading to a confluent cells network covering almost the entire surface of the nanostructured rGO microfiber, as shown in Fig. 4d, which obviously presrnt a larger coverage compared with the 10-day’s result. The higher resolution SEM images of the cells are also shown in Fig. 4b1, c1 and d1, the cells density is increasing by time, cells were tighter packed from day 5 to day 10, and multilayered filopodia of cells can be seen at day 15. The result demonstrates that the nanostructured rGO microfiber can provide a favorable substrate for cell adhesion and spreading. The nanoporous topography of the nanostructured rGO microfiber surface is believed to ensure the efficient mass transport of nutrition for NSCs metabolic demands, which should facilitate cells adhesion and growth^23^. The SEM observation of NSCs spreading at different times demonstrates that the nanostructured rGO microfiber could be a powerful substrate for NSCs.

### Differentiation of NSCs on the scaffolds

**Figure 5:**
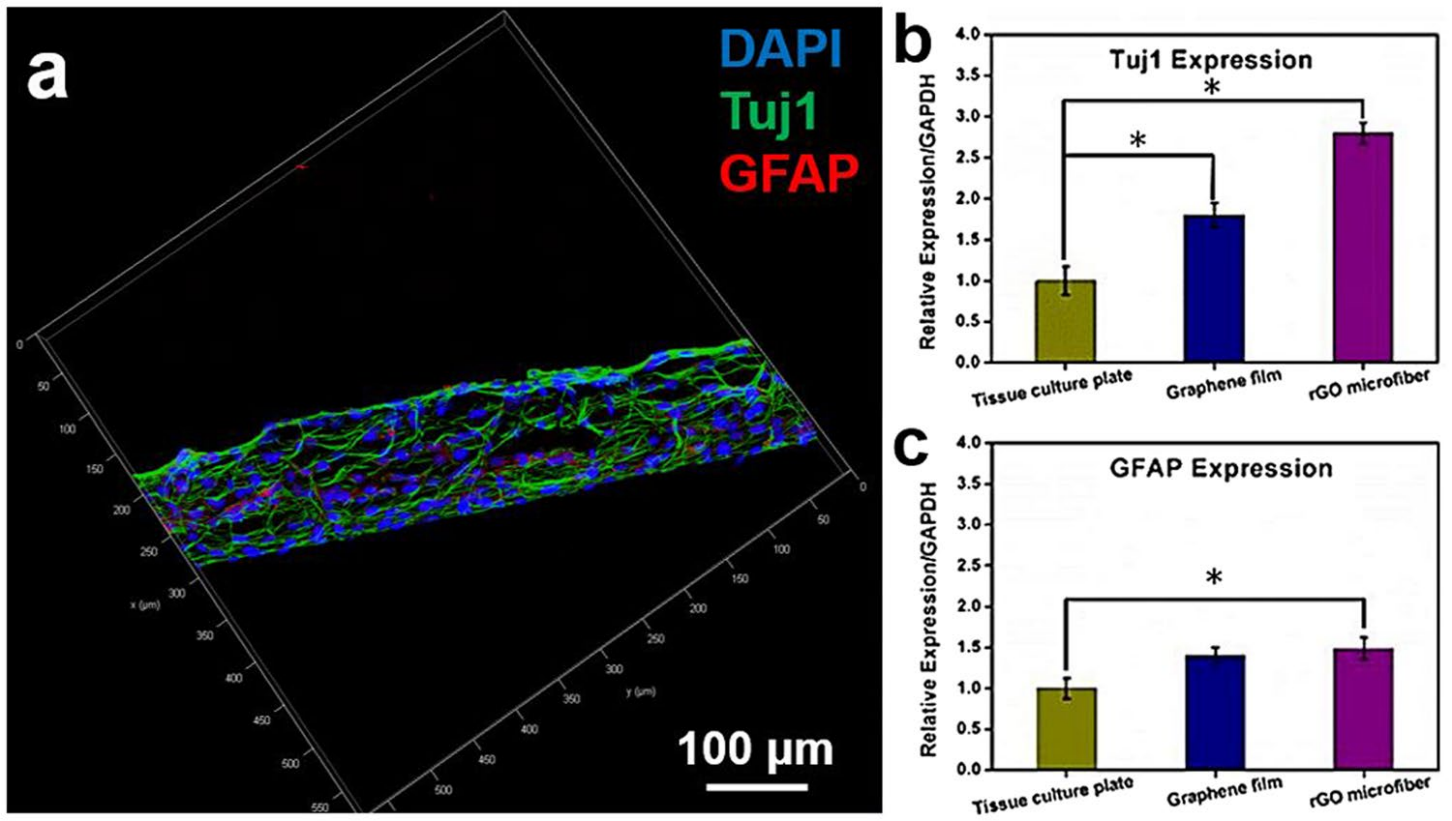
The immunostaining of NSCs on nanostructured rGO microfiber and the qPCR analysis of NSCs on tissue culture plate, 2D graphene film and nanostructured rGO microfiber. (**a**) NSCs were immunostained with neural-specific antibodies after cultured under differentiation condition for 15 days. CLSM fluorescent micrographs of the cells immunostained with DAPI for nuclei (blue) and Tuj1 for neuron (green), GFAP for glia (red). (Scale bar = 100 μm) (**b**) (**c**) refer to the qPCR analysis of the cells genes expression levels of Tuj1 and GFAP on tissue culture plate, 2D graphene film and nanostructured rGO microfiber for 15 days under neural differentiation conditions. (*р ≤ 0.05, n = 3).

To evaluate the neural differentiation states of NSCs on nanostructured rGO microfibers, NSCs cultured on the fibers for 15 days were studied by immunostaining, and the differentiation period was preceded by a 5 day proliferation phase. During the NSCs differentiation, some of the cells differentiate into neurons, whereas others may differentiate into glia cells, which support the nervous tissue and neuron activity. From the previous study to use NSCs for neural regeneration, it is critical to induce NSCs differentiation that is directed more toward neurons than glial cells^24^. For this purpose, after the cells were cultured on the nanostructured rGO microfiber under neural differentiation condition for 15 days, the neurons and glial cells differentiated from NSCs were marked by Tuj1 (green) and GFAP (red)^25^. From the immunostaining micrographs (Fig. 5a), cells were well attached on the fibers during the differentiation period, a majority of cells were Tuj1 positive, but a few of cells were GFAP positive. These results indicating that NSCs keep the pluripotency to differentiate into both neuronal subtypes, and the cells seem more favorable differentiated into neurons toward glia cells on the nanostructured rGO microfiber. NSCs differentiated on tissue culture plate and 2D graphene film were also investigated. After the cells were cultured on tissue culture plate for 15 days under differentiation conditions, Tuj1 positive cells (Fig. S12a) were much less than GFAP positive cells. On the 2D graphene film (Fig. S12b), Tuj1 positive cells significantly more than that on the tissue culture plate, the GFAP staining also indicate that GFAP expression was more active on 2D graphene film. Some previous studies reported that the graphene-based scaffolds worked as excellent cell-adhesion substrates during the long-term differentiation process and induced the differentiation of NSCs more toward neurons than glial cells^24^, but the mechanism was not clearly. These findings suggest that the nanostructured rGO microfiber and 2D graphene film are more powerful substrates for NSCs neural differentiation than the cells on tissue culture plate. The 3D structure micrographs and Movie S2 indicate that the cells could uniformly spread around the graphene fiber, providing a guidance scaffold for the growth of NSCs and forming a dense neuronal network.

For investigating the neuronal differentiation tendency of NSCs on the nanostructured rGO microfiber, the NSCs genes expression levels of neuronal-specific makers at day 15 were evaluated. The qPCR assay was carried out for detecting the gene expressions of Tuj1 and GFAP. For comparison, cells Tuj1 and GFAP expressions on tissue culture plate and 2D graphene film were also investigated. As shown in Fig. 5b, compared with the cells that cultured on the tissue culture plate, the expression of Tuj1 of NSCs cultured on the 2D graphene film was enhanced in cells by ~1.8-fold, and ~2.8-fold on the nanostructured rGO microfiber. The expressions of GFAP (Fig. 5c) of the NSCs on 2D graphene film and nanostructured rGO microfiber were enhanced by ~1.4-fold and ~1.43-fold compared with the cells on tissue culture plate, respectively. These results indicate that graphene substrates are benefit for the NSCs neuronal differentiation, and it also demonstrated that the nanostructured rGO microfiber can act as a more favorable substrate for the enhancement of NSCs differentiation into neurons over that into glia cells than 2D graphene film.

From the above results, the nanostructured rGO microfiber shows excellent properties, which could match the demands of NTE scaffolds with appropriate mechanical properties, cytocompatibility, release of non-toxic degradative products, appropriate structures for cells proliferation, adhesion and spreading, and provide favorable substrate for NSCs neural differentiation.

The cells proliferation, adhesion, cytocompatibility and other biological effects of a scaffold are critical properties to be considered for tissue engineering applications^26^. Nerve cells are anchorage-dependent cells, which require sufficient adhesion to a substrate to spread, proliferate and maintain cellular functions^27^. The ripples and nanoporous topography of the surface of nanostructured rGO microfiber probably resulted in improved mechanical interconnections at the cell-material and cell-cell interfaces^28^, which consequently cause a better cellular adhesion effect than 2D graphene film. In this study, no obvious cytotoxicity of the nanostructured rGO microfiber was observed for NSCs. The nanostructured rGO microfiber for NSCs was better able to maintain a larger immature (Nestin+) cell population than that of the 2D graphene film.

Apart from the high cytocompatibility, the nanostructured rGO microfiber can regulate the NSCs differentiation toward neurons over glia cells. The enhancement may certainly be caused by the complex interactions of the chemical and physical properties of the cell-cell and cell-material interfaces^29^. Meanwhile, the flexibility, stiffness, dimensions and morphologies of the scaffolds could be the biological effects that regulate the fate of stem cells. For example, the fiber-shaped channel structure could confine the NSCs spreading and facilitate the formation of a neuronal network around it. The nanoporous structure of the surface of the nanostructured rGO microfiber provide a better platform for cellular communication, migration of nutrients and cellular metabolism.

Altogether, these results demonstrate the versatility of such nanostructured rGO microfiber scaffolds in regulating NSCs neural differentiation through a combination of topographical and biochemical signaling and illustrate the great potential of the nanostructured rGO microfiber as an artificial NTE scaffold for nerve regeneration.

## Conclusion

In summary, we introduce a novel utilization of a nanostructured rGO microfiber as a light, flexible, mechanical strong and cytocompatible scaffold for NSCs culturing *in vitro*. With its excellent cytocompatibility and protein adsorption ability, the nanostructured rGO microfiber can not only support NSCs growth but also keep the cells at a more active differentiation state with Tuj1 expression while inhibiting the expression of GFAP. The experimental results prove that the nanostructured rGO microfiber can regulate the NSCs differentiation into neurons. Furthermore, the NSCs can form a dense neural network around the nanostructured rGO microfiber, just like a functional nerve graft. Our findings implicate the great potential of nanostructured rGO microfibers as an artificial NTE scaffold for nerve regeneration.

## Experimental Section

### Preparation of graphene oxide

The GO was prepared by the modified Hummers’ method^30^. Briefly, graphite powder was pre-treated with concentrated H_2_SO_4_, K_2_S_2_O_8_ and P_2_O_5_. Then, the pre-treated graphite powder was oxidized with concentrated H_2_SO_4_ and KMnO_4_ via the traditional Hummers’ method.

### Preparation of nanostructured rGO microfiber

The nanostructured rGO microfiber was produced by a capillary hydrothermal method. The GO suspension (dissolved in 10% ethanol solution) with a concentration of 8 mg/ml was injected into a glass pipeline with a 1.0 mm inner diameter that was sealed up on both ends (see Fig. S2a) and maintained in an oven at 220 °C for 6 h. nanostructured rGO microfiber samples matching the pipeline geometry were thereby obtained (Fig. S2b,c).

### Characterization of nanostructured rGO microfiber

The topography of the samples was characterized by a SEM (SU8020, Hitachi, Japan). A Dilor XY microspectrometer with 532 nm laser excitation was used to record the Raman spectra of the graphene fiber. The mechanical properties were analyzed by an Instron material testing system (Instron 3365/series IX/s). The room temperature electrical property was measured by a semiconductor characterization system (Keithley 4200-SCS).

### Cell culture

NSCs were isolated and purified from the cerebral cortex of a rat embryo, and the detailed information is presented in supporting information S8. Passage 2 of the isolated cells was used in the experiments. For proliferation studies, passage 2 NSCs were seeded at a density of 30000 cells/cm^3^ in proliferation culture medium containing DMEM-F12 (Life Technologies, USA) with 2% B27 (Life Technologies, USA), 1% N2 supplement (Life Technologies, USA), EGF (20 ng/ml, PeproTech, USA), bFGF (20 ng/ml, Pepro Tech, USA) and 1% penicillin-streptomycin (Life Technologies, USA). Differentiation of the NSCs was induced by exchanging the proliferation medium containing 1% fetal bovine serum (FBS, Life Technologies, USA) and withdrawing the growth factors.

To ensure the adhesion of the cells, the nanostructured rGO microfibers (or 2D graphene film) were incubated in 2 ml 10 ng/ml poly-D-lysine (PDL, Sigma, USA) solution overnight in the incubator, washed with PBS, and then incubated in 1 ml 10 ng/ml laminin (Life Technologies, USA) solution for 6 h, the fibers were moved to no-coating culture plate before cells seeding. NSCs were seeded at a density of 30000 cells/cm^3^ and cultured under proliferation or differentiation conditions.

The fibers were tailored and full-filled the bottom of a well of 24-well plate, and cells seeding density was calculated by the volume of the fibers. The area of graphene film was the same with the well of 24-well plate, and the cells seeding density was also the same with the fibers no matter whether had fibers. The tissue culture plate and 2D graphene film were also coated with PDL and laminin.

### Cell viability assay

To assess the viability of the NSCs on nanostructured rGO microfibers, NSCs were cultured on nanostructured rGO microfibers for 3 days under proliferation conditions, and the viability state of the cells was checked by staining the cells with the LIVE/DEAD Cell Imaging kit for mammalian cells (Life Technologies, USA) according to the manufacturer’s instructions.

A Cell Count Kit-8 (CCK-8, Dojindo Molecular Technology) was used to quantitatively evaluate cell viability on tissue culture plates, graphene films and nanostructured rGO microfibers after cultivation for 1, 3 and 5 days.

To further demonstrate the cytocompatibility of the nanostructured rGO microfibers, after the NSCs were cultured for 5 days under proliferation condition, the cells were fixed with 4% paraformaldehyde, permeabilized with 0.1% Triton X-100, and blocked with 1% bovine serum albumin (BSA, Sigma, USA) solution at room temperature. To check the stemness of the NSCs and the location and distribution of the cells, the fixed cells were incubated with Alexa Fluor 488 conjugated Nestin (1:100, Millipore, USA) for 60 min and the nuclei were stained by 4′ 6-diamidino-2-phenylindole (DAPI, 300 nM, Life Technologies, USA) for 10 min. Finally, the cells were washed with TPBS three times before being examined with a Leica Confocal Microscope SP8.

### SEM observation of NSCs cultured on nanostructured rGO microfibers

The morphologies of the cells cultured on nanostructured rGO microfibers after 5, 10 and 15 days under proliferation conditions were observed by a SEM (Hitachi; SU8020). The cells seeded on nanostructured rGO microfibers were removed from the culture medium and gently washed with Dulbecco’s phosphate-buffered saline (DPBS). Cells on the scaffolds were fixed with 4% paraformaldehyde in PBS for 2 h at room temperature. After removing the fixative, the scaffolds were gently washed with PBS and immersed in 1% w/V osmic acid for 2 h. After washing, these microfiber samples were subjected to sequential dehydration for 15 min twice each with an ethanol series (30%, 50%, 70%, 85%, 90%, 95%, 98% and 100%). After lyophilization and coating with gold, the scaffolds with cells were observed under the SEM to assess the cell attachment and morphology at a 5 kV accelerating voltage.

### Assessment of the differentiation of NSCs on nanostructured rGO microfibers by the immunochemistry method

To assess the neural differentiation of NSCs on nanostructured rGO microfibers, NSCs were cultured on the microfibers 15 days under differentiation conditions, which was prior to a 5 days proliferation phase. The cells were washed with PBS, fixed in 4% paraformaldehyde for 60 min, extracted with 0.1% Triton X-100 (Sigma, USA) for 10 min and blocked with 10% goat serum (Sigma, USA) for 2 h. The primary antibodies were incubated overnight at 4 °C, and the secondary antibodies were incubated for 2 h at room temperature, followed by DAPI staining; the primary antibody panel used included primary antibodies against Tuj1 (1:500, Abcam, USA) and GFAP (1:1000, Abcam, USA), and the second antibody used was Alexa Fluor 488-conjugated goat anti-mouse or anti-rabbit IgG (1:200, Jackson ImmunoResearch, USA) for staining and evaluation of the expression of characteristic proteins of neurons and glial cells. A Leica Confocal Microscope SP8 was used to observe the protein expressions of the stained cells.

### Assessment of the differentiation of NSCs on nanostructured rGO microfibers by qPCR

NSCs differentiation on nanostructured rGO microfibers was also assessed by the gene expressions using qPCR at day 15. The total RNA was extracted from the cells using the RNeasy Plus Mini Kit (Qiagen), according to the manufacturer’s instructions. The RNA samples were reverse-transcribed into cDNA for qPCR using the PrimeScriptTM reagent kit with gDNA Eraser (Takara). qPCR was performed with SYBR Premix Ex TaqTM with ROX (Takara), according to the manufacturer’s instructions. The signals were detected with an ABI 7500 Fast Real Time PCR system (Applied Biosystems) to analyze the expressions of Tuj1 and GFAP. The gene expression was normalized to glyceraldehyde-3-phosphate dehydrogenase (GAPDH) as the internal standard. Information on the primers is provided in Supplementary Information Table S1.

### Statistical analysis

The data were reported as the means ± SD, and statistical analysis was performed using the unpaired Student’s t-test. Statistical significance was accepted at *р ≤ 0.05.

## Electronic supplementary material

Supporting Information

Movie 1

Movie 2
