# Supplementary material for: Graphene microfiber as a scaffold for regulation of neural stem cells differentiation
Source: Sci Rep. 2017 Jul 18;7:5678. doi: 10.1038/s41598-017-06051-z (PMC5515841; doi:10.1038/s41598-017-06051-z)
Supplement: Supplementary file 1 — Supporting Information [file 41598_2017_6051_MOESM1_ESM.doc]

**Supporting Information**

Graphene microfiber as a scaffold for regulation of neural stem cells differentiation

**Weibo Guo,^1^ Jichuan Qiu,^2^ Jingquan Liu,^*1,3^ Hong Liu^*2^**

^1^Shandong Province Key Laboratory of Detection Technology for Tumor Makers, College of Chemistry and Chemical Engineering, Linyi University, Linyi 276005, P. R. China.

^2^State Key Laboratory of Crystal Materials, Shandong University, Jinan, 250100, P. R. China.

^3^College of Materials Science and Engineering, Laboratory of Fiber Materials and Modern Textile, The Growing Base for State Key Laboratory, Qingdao University, Qingdao 266071, P. R. China.

Corresponding Authors: [hongliu@sdu.edu.cn](mailto:hongliu@sdu.edu.cn); jliu@qdu.edu.cn

**This PDF file contents including as follows:**

**S1: Atomic Force Microscopy (AFM) of GO nanosheets**

**S2: Real photographs of preparation of rGO nanostructured microfiber**

**S3: Raman spectrum of rGO microfiber before drying.**

**S4: Zeta potential of rGO.**

**S5: XPS characterization for O/C ration of rGO nanostructured microfiber.**

**S6: BCA test of adsorption of BSA on 2D graphene film and rGO nanostructured microfiber**

**S7: Characterization of isolated NSCs**

**S8: Characterizations of 2D multilayer graphene film.**

**S9: Cell viability assays of NSCs on tissue culture plate**

**S10: Cell viability assays of NSCs on 2D graphene film**

**S11: Nestin staining of NSCs proliferated on tissue culture plate and 2D graphene film for 5days**

**S12: Differentiation of NSCs on tissue culture plate and 2D graphene film for 15 days**

**S13: Sequences of Real-Time PCR primers**

**S14: Captions for supporting Movies**

**S1: Atomic Force Microscopy (AFM) of GO nanosheets**


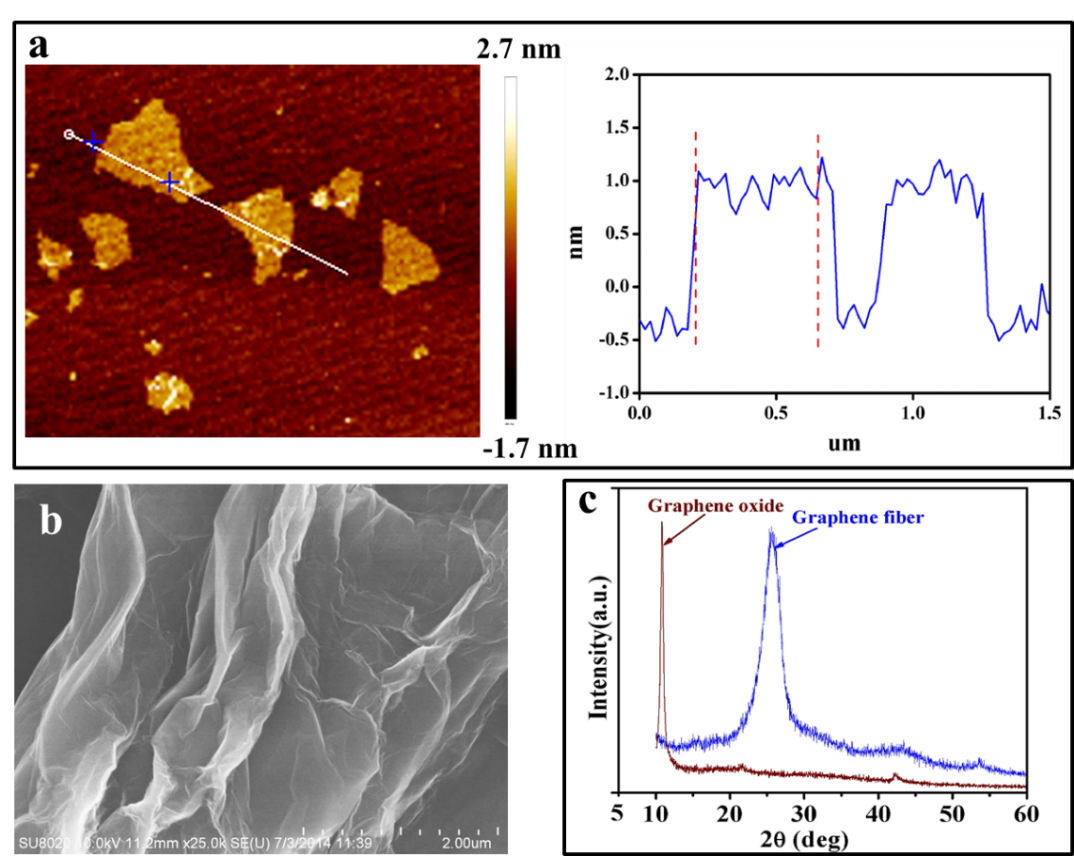


Figure S1 Atomic Force Microscopy (AFM) of GO nanosheets.

**S2: Real photographs of preparation of rGO nanostructured microfiber**


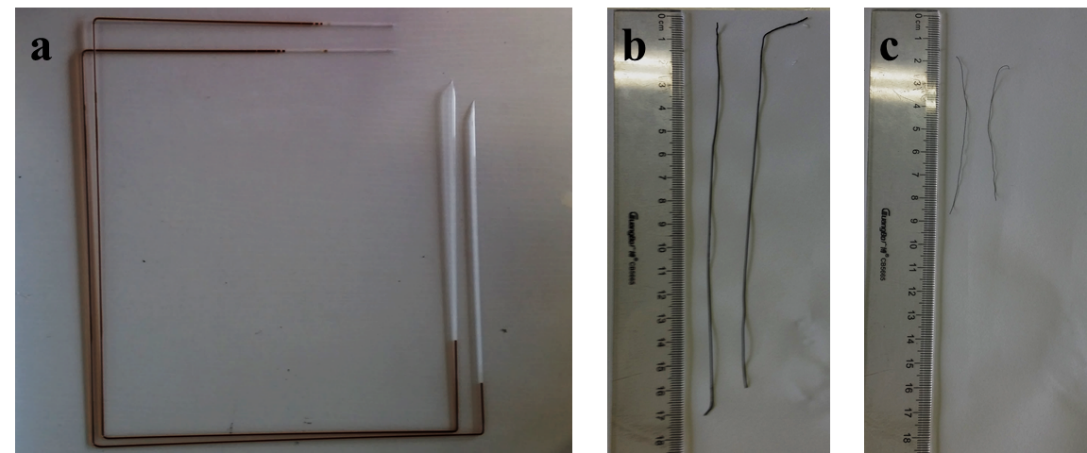


Figure S2 (a) Photograph of about 1 m long glass pipelines (1.0 mm in inner diameter) filled with 8 mg/ml aqueous graphite oxide (GO) suspension. Photographs of obtained rGO nanostructured microfiber before (b) and after (c) natural drying.

**S3: Raman spectrum of rGO microfiber before drying**


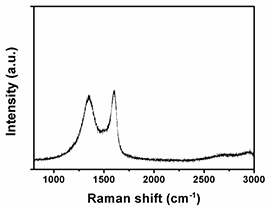


Figure S3 Raman spectrum of rGO microfiber before drying.

**S4: Zeta potential of rGO**

**
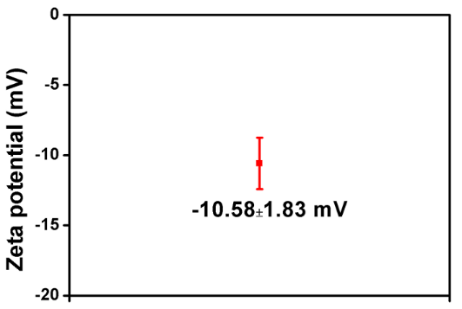
**

Figure S4 Zeta potential of rGO.

**S5: XPS characterization for O/C ration of rGO nanostructured microfiber**

**
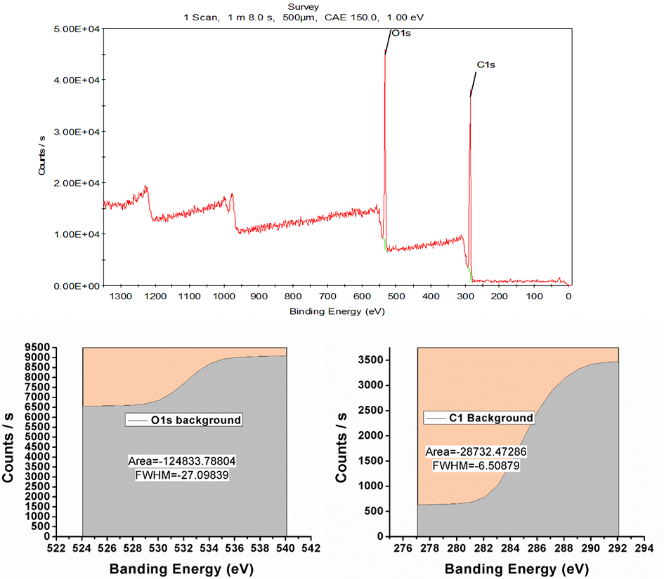
**

Figure S5 XPS spectrum of rGO nanostructured microfiber

**S6: BCA test of adsorption of BSA on 2D graphene film and rGO nanostructured microfiber**

**
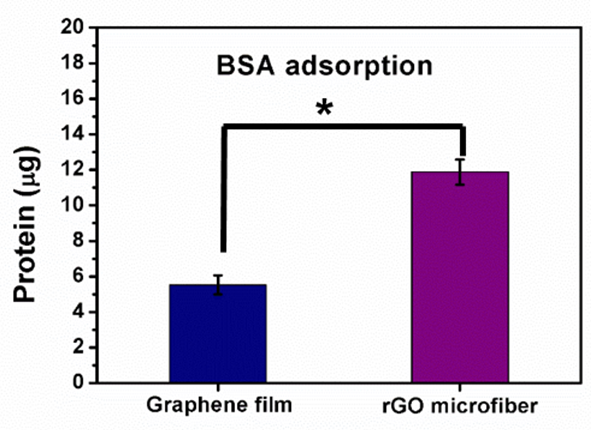
**

Figure S6 Adsorption of BSA on 2D graphene film and rGO nanostructured microfiber. (*р≤0.05, n=3)

**S7: Characterization of isolated NSCs**


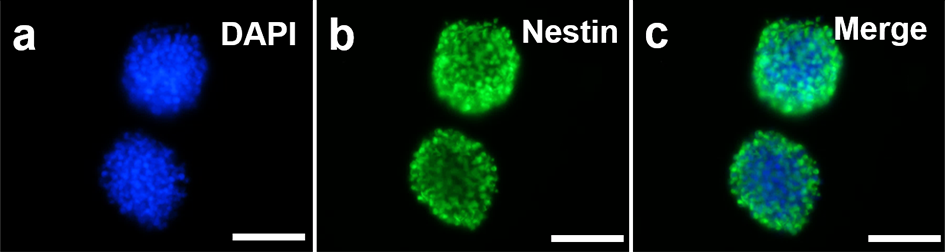


Figure S7 Nestin immunostaining of the isolated NSCs, the suspension cultured NSCs were stained with Nestin (green) (b) and the nuclei stained with DAPI (blue) (a), (c) is the merged fluorescence micrographs. (Scale bars=100 μm)

NSCs were isolated from the cerebral cortex of rat embryo, which was taken out from an embryonic day 13 (E13) SD pregnant rat, suspension cultured with proliferation culture medium, and evaluated by Nestin staining.

In order to identify the isolated NSCs, the process of NSCs suspension immunostaining as follows: (1) Collect and rinse the cells with Phosphate Buffered Saline (PBS) once; (2) Fixe the cells with 4 % paraformaldehyde at 4 °C for 15 min; (3) Wash twice with the 0.5 % bovine serum albumin (BSA) containing PBS and 0.1 % saponin (Buffer A) twice; (4) Resuspend the cells in small volume of Buffer A, add 1ug Nestin antibody, incubate for 1hour at 4 °C; (5) Wash twice with Buffer A, and then staining DAPI for 15 min at room temperature; (6) Wash twice and keep the cells in 0.5 % BSA (Buffer B) containing PBS at 4 °C in dark. Nestin, an intermediate filament protein, which is the typically expressed protein for NSCs. The results showed that almost NSCs cultured in suspension are Nestin positive.

**S8: Characterizations of 2D multilayer graphene film**


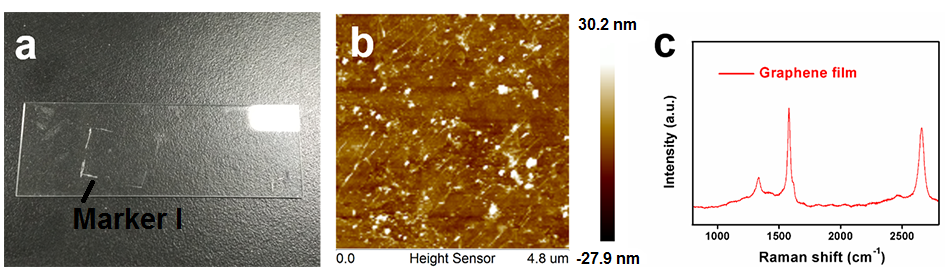


Figure S8 (a) Digital photograph of few-layer graphene on the glass substrate; (b) Atomic Force Microscopy (AFM) of the graphene film; (c) Raman spectrum of graphene film.

**S9: Cell viability assays of NSCs on tissue culture plate**


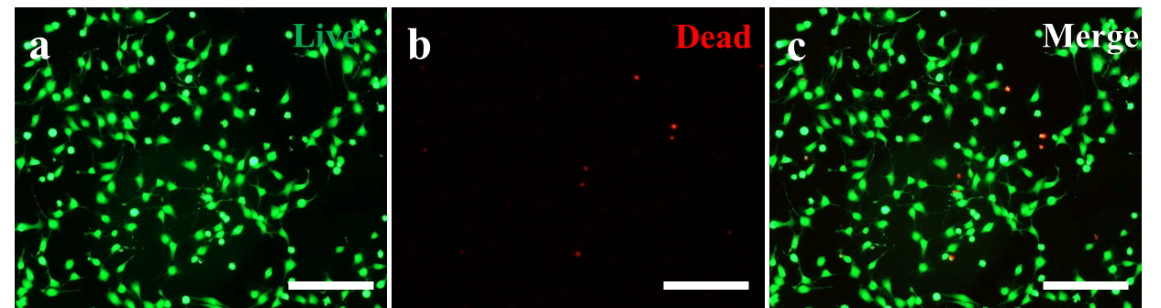


Figure S9 Cell viability assay of NSCs on tissue culture plate after 3 days culture as determined by Live/Dead assay, live cells were stained green (a) and dead cells were stained red (b), (c) is the merged fluorescence micrographs. (Scale bars=100 μm)

**S10: Cell viability assays of NSCs on 2D graphene film**


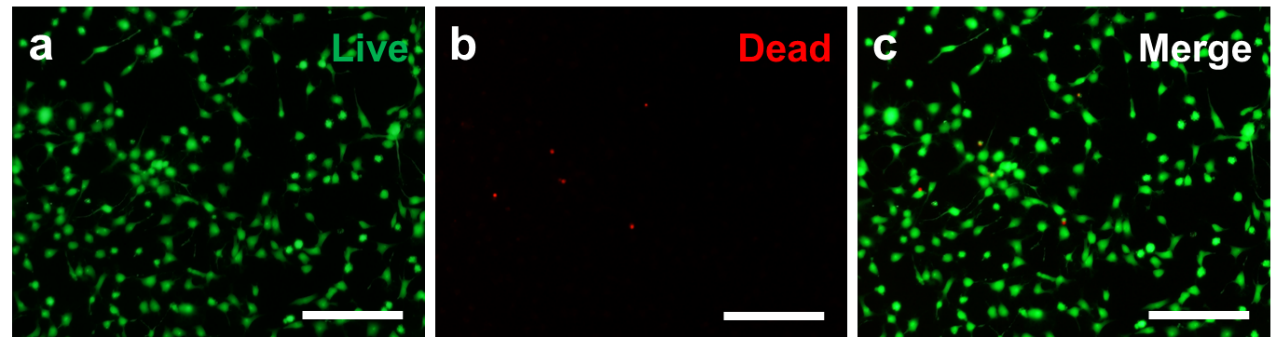


Figure S10 Cell viability assay of NSCs on 2D graphene film after 3 days culture as determined by Live/Dead assay, live cells were stained green (a) and dead cells were stained red (b), (c) is the merged fluorescence micrographs. (Scale bars=100 μm)

**S11: Nestin staining of NSCs proliferated on tissue culture plate and 2D graphene film for 5days**


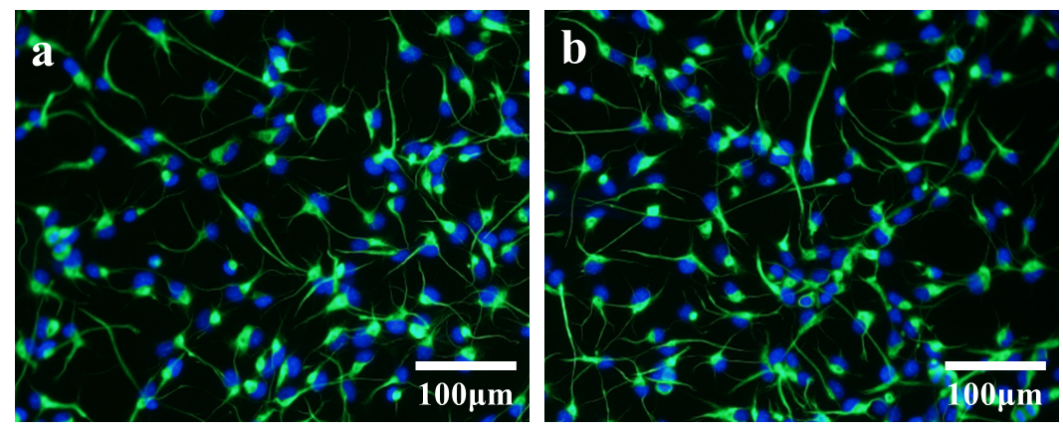


Figure S11 CLSM fluorescence micrographs of NSCs proliferated on tissue culture plate (a) and 2D graphene film (b) for 5 days, immunostaining makers were DAPI (blue) for nuclei and Nestin (green) for NSCs.

**S12: Differentiation of NSCs on tissue culture plate and 2D graphene film for 15 days**


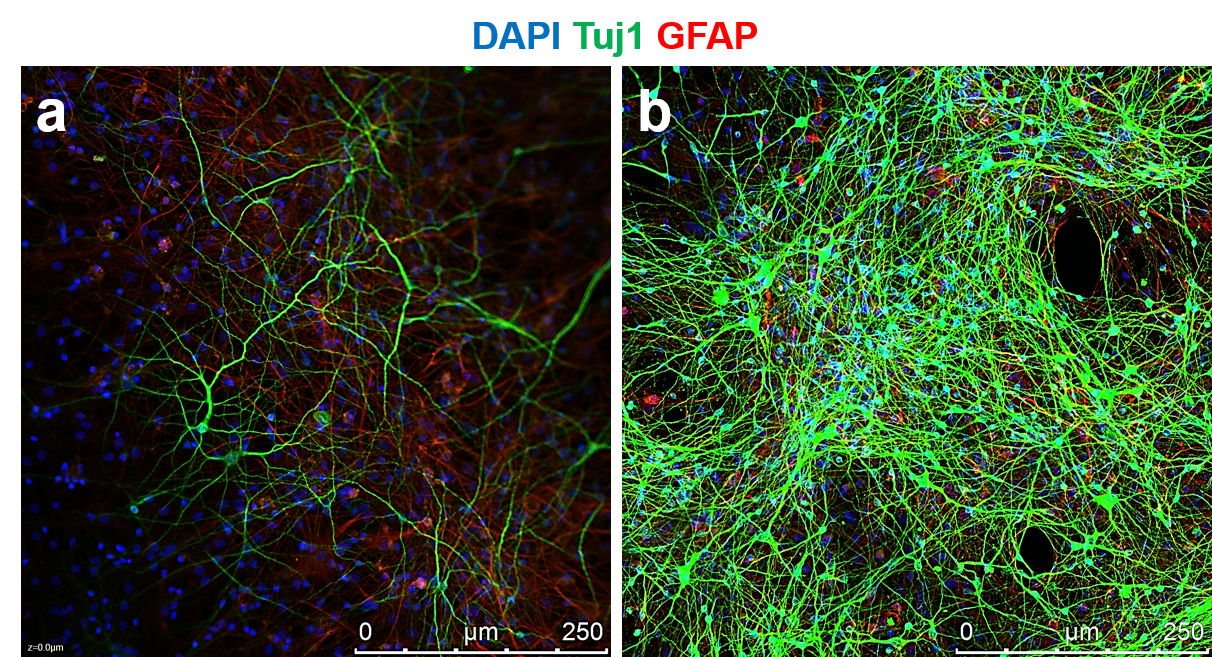


Figure S12 NSCs were immunostained with anti-Tuj1 (green), anti-GFAP (red) antibodies after the cells were cultured on tissue culture plate (a) and 2D graphene film (b) for 15 days under differentiation condition, and the nuclei were stained with DAPI (blue).

**S13:** Sequences of Real-Time PCR primers

| Gene | Forward primers (5'-3') | Reverse primers (5'-3') |
| --- | --- | --- |
| GAPDH | GCCTCGTCTCATAGACAAGATGGT | GAAGGCAGCCCTGGTAACC |
| Tuj1 | TAGACCCCAGCGGCAACTAT | GTTCCAGGCTCCAGGTCCACC |
| GFAP | CGGAGACGTATCACCTCTG | TGGAGGCGTCATTCGAGACAA |

Table S1 Sequences of Real-Time PCR primers

**S14: Captions for supporting Movies**

**Movie S1:** This movie shows the rotation video of the NSCs cultured on rGO nanostructured microfiber for 5 days and stained with Nestin (green) and DAPI (blue).

**Movie S2:** This movie shows the rotation video of NSCs differentiated on rGO nanostructured microfiber for 15 days and immunostained with Tuj1 (green), GFAP (red) and DAPI (blue).
